# Case of Addison’s or Bronzed Disease

**Published:** 1868-01

**Authors:** A. Young

**Affiliations:** Prescott, Illinois


ARTICLE III.

### CASE OF ADDISON'S OR BRONZED DISEASE.

REPORTED BY A. YOUNG, M.D., Prescott, Illinois.

As the etiology of this disease is still involved in obscurity, any facts bearing upon it may be of interest to the profession. This iuduces me to give a brief resume of a case which was recently under my care.

Rev. Mr. R., aged 46, had for several years suffered from ill health, which was, however, not sufficient to interrupt his professional duties, except by brief intervals. As there was evidence of tubercular deposits in both lungs, his ill health was attributed to this cause. Last winter his general health began to fail; there was some disturbance of the digestive organs, but no apparent increase of the disease of the lungs. About the same time, the skin of the face, neck, and hands, which was naturally rather dark and sallow, began to assume a darker hue, and eventually became as intense as that of a jaundiced person, except that the hue had not the yellow tinge of jaundice, but more nearly resembled a person deeply tanned by exposure. The debility increased, until he was obliged to relinquish his occupation, but was able to be around, complaining of nothing but extreme lassitude and weakness, till September 16th, when persistent nausea and vomiting, with increase of prostration and faintness, supervened, continuing till his death, which occurred September 23d.

A *post-mortem* revealed crude, tubercular deposits in apex of both lungs, some fatty degeneration of heart.

Abdominal viscera presenting nothing worthy of note, except kidneys, and supra-renal capsules. Left kidney healthy in structure, with the exception of a small tubercular deposit. Left supra-renal cupsule filled with crude tubercle. The natural texture of the right kidney and supra renal cupsule entirely destroyed, its place being filled by a soft pultaceous mass, undoubtedly softened tubercle.

During the time that I knew Mr. R., there was nothing to indicate renal disease, the urine being normal in quantity and quality.

